# Atypical Myocardial Infarction with Apical Thrombus and Systemic Embolism: A Rare Presentation of Likely JAK2 V617F-Positive Myeloproliferative Neoplasm

**DOI:** 10.1155/2020/9654048

**Published:** 2020-05-19

**Authors:** Muhammed Atere, Rana Al-Zakhari, Jennifer Collins, Francesco Rotatori, Lloyd Muzangwa

**Affiliations:** Richmond University Medical Center, USA

## Abstract

A few types of myeloproliferative neoplasms may be significant for Janus-associated kinase 2 mutation, JAK2 V617F, including polycythemia vera, essential thrombocythemia, and primary myelofibrosis. The prevalence of JAK2 mutation is low in the general population but higher in patients with myeloproliferative neoplasms. Some patients with JAK2 V617F-positive essential thrombocythemia are asymptomatic, but others may develop hemorrhagic or thromboembolic complications. Thromboembolism may occur in vessels of high flow organs like the heart and, thereby, present as myocardial infarction. Nonetheless, these patients are usually symptomatic with complaints of chest pain, for example. Atypical (asymptomatic) myocardial infarction with mild thrombocytosis may be the first clue for possible essential thrombocythemia with JAK2 V617F. In this report, we discuss a case of atypical (asymptomatic) myocardial infarction with secondary thromboembolism in a patient positive for JAK2 V617F with a likely myeloproliferative neoplasm.

## 1. Introduction

Myeloproliferative neoplasms (MPNs) are majorly classified into chronic myeloid leukemia (CML), polycythemia vera (PV), essential thrombocythemia (ET), and primary myelofibrosis (PMF) [1]. Other minor subtypes are chronic neutrophilic leukemia (CNL), hypereosinophilic syndrome (HES), and chronic eosinophilic leukemia (CEL) [1]. Janus-associated kinase 2 (JAK2) is a protein that acts as an enzyme in the transfer of gamma phosphate in adenosine triphosphate to hydroxyls of tyrosine residues [2]. Its mutation, JAK2 V617F, has been linked to MPNs, including ET, PV, and PMF [1–4].

JAK2 V617F prevalence may vary by population. In a study by Syeed, 74% (67) of 90 Kashmiri patients with MPNs tested positive for JAK2 V617F [2]. In another article by Da Silva et al., 65% (93) of 144 patients with MPNs in Pernambuco, Brazil, were positive for JAK2 V617F [3]. The mutation prevalence among persons with MPN was about 58% (64) out of 110 individuals at the National Cancer Institute, Cairo University [4]. However, the prevalence appears to be lower in the general community. In a published article by Nielsen et al. involving Copenhagen population of 49,488, just 63 (approximately 0.1%) tested positive for JAK2 V617F [5]. MPNs are characterized by clonal proliferation of one or more types of cells of myeloid series with an increased number of progenitor cells of myeloid lineages in the bone marrow (BM) and immature and mature cells in the peripheral blood [1, 6].

Symptoms may be similar, but there are still variations depending on whether patients have ET, PV, or MP [7]. Other patients may be asymptomatic until the development of complications. Thromboembolism is a documented complication that causes occlusions in the vessels of individual organs. Thromboembolism may develop in both arterial and venous systems, particularly at the time or after diagnosis [8]. Hemorrhagic complications from acquired Von Willebrand syndrome may also be a feature of ET [9]. Here, we present a patient with possible JAK2 V617F-positive MPN who had atypical ST-elevation myocardial infarction (STEMI) and a cardiac apical thrombus with systemic embolism.

## 2. Case Report

**Figure 1:**
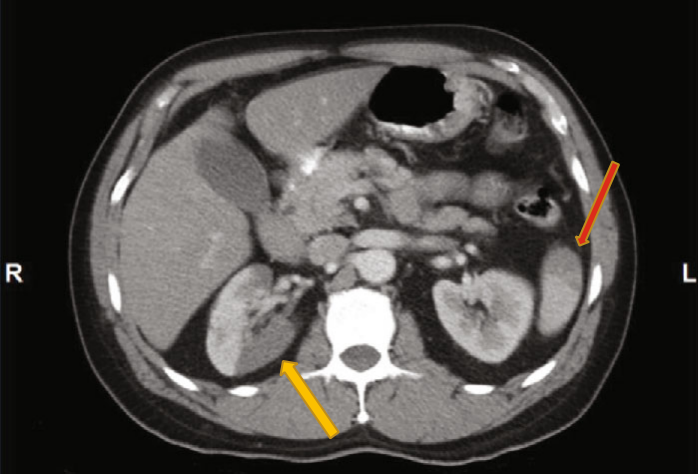
A CT scan of the abdomen: yellow arrow - right renal infarction; red arrow - splenic infarction.

**Figure 2:**
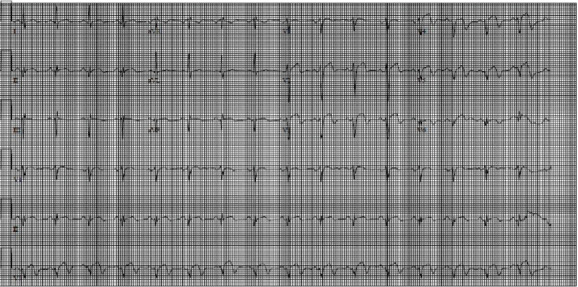
An electrocardiogram: left axis deviation, ST segment elevation in V2 to V5, and Q waves in inferior leads.

A 58-year-old man with no significant medical history presented to the emergency room for evaluation of right-sided abdominal pain of two days. He denied chest pain, shortness of breath, or palpitations. Physical examination was essentially normal. A computed tomography (CT) was done to rule out an acute intra-abdominal pathology; however, it revealed areas of infarctions in the right kidney and spleen (Figure 1). An electrocardiogram (EKG) demonstrated left axis deviation, ST-segment elevation in V2 to V5, and Q waves in inferior leads signifying a recent inferior-apical infarct (Figure 2). Laboratory investigations on day 0 showed troponin of 12.6 ng/mL, a white blood cell count of 13,900/*μ*L, a platelet count of 540,000/*μ*L, and a hemoglobin concentration of 15 gm/dL. Furthermore, a liver function was within a normal limit except for an aspartate transaminase of 80 U/L (normal range: 15–37 U/L), and urinalysis was insignificant for an infection or hematuria. He was started on a heparin continuous infusion and administered aspirin tablet 325 mg and ticagrelor 180 mg.

**Figure 3:**
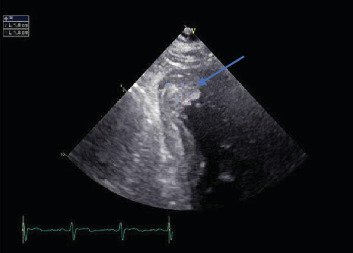
An echocardiogram: blue arrow - a mass suggestive of a thrombus.

**Figure 4:**
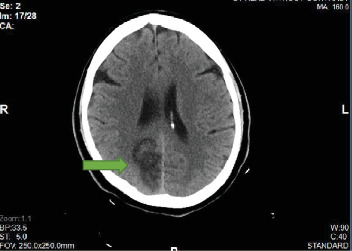
A CT head without contrast (during initial hospitalization): green arrow - a subacute right occipital infarct.

On day 1, the platelet count dropped to 461,000/*μ*L, but it began rising slowly to 789,000/*μ*L on day 7. On day 8, the platelet count decreased mildly to 738,000/*μ*L but rose to a peak of 919,000/*μ*L on day 11 and then decreased to 788,000/*μ*L on day 13, the day he was discharged. Hemoglobin remained within normal limits during hospitalization. An echocardiogram done on day 1 demonstrated an estimated left ventricular ejection fraction of 60-65% and severe apical hypokinesis with a 1.8 × 1.0 cm mass suggestive of a thrombus (Figure 3). He had a coronary angiogram on day 2, which revealed a total occlusion in the distal left anterior descending coronary artery. We became suspicious of a hypercoagulable state because of a persistently high platelet count. A peripheral blood smear was significant for large platelets. The hypercoagulable workup showed that at least one allele was positive for JAK2 V617F. Other hypercoagulability investigations including anti-neutrophilic antibody, cardiolipin antibody, antiphospholipid, lupus anticoagulant, factor V Leiden antibody, and prothrombin G20210A were all unremarkable. A BM biopsy was planned, but he declined. The patient was clinically stable until before a planned discharge when he developed numbness in the left face prompting a CT head to be done, which showed a subacute right occipital infarct (Figure 4).

**Figure 5:**
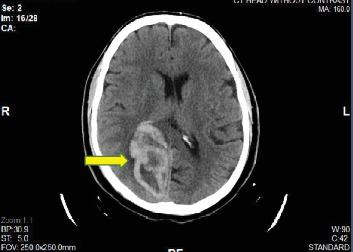
A CT head without contrast (two days after discharge): yellow arrow - a hemorrhagic right occipital infarct.

He was eventually discharged after stability, and a therapeutic international normalized ratio between 2 and 3 was achieved. His discharged medications were aspirin (81 mg/day), warfarin (initial dose of 5 mg/day) with heparin infusion drip as a bridge, metoprolol tartrate (25 mg twice daily), atorvastatin (80 mg/day), and lisinopril (5 mg/day). He presented two days later with a headache. A CT scan of the head done showed hemorrhage in the right occipital lobe with surrounding vasogenic edema, consistent with a hemorrhagic infarct as well as a mass effect on the right occipital horn (Figure 5). He continued to receive care while hospitalized, but he signed against medical advice about three days later. Unfortunately, he never followed up as an outpatient.

## 3. Discussion

ET is defined as a myeloproliferative clonal disease resulting in megakaryocyte expansion in the bone marrow and an increased number of platelets in the peripheral blood [1, 6]. The revised World Health Organization (WHO) criteria used to diagnose this condition are four major criteria and one minor criterion. The major criteria include

- a platelet count greater than or equal to 450,000/L [10]
- a BM biopsy demonstrating the proliferation of platelets [10]
- not classified under BCR-ABL1+ CML, PV, PMF, myelodysplastic syndromes, or other myeloid neoplasms [10]
- the presence of JAK2, calreticulin, or myeloproliferative leukemia virus oncogene mutation [10]

The minor criterion is the presence of a clonal marker or absence of reactive thrombocytosis [10]. Any patient with all four major criteria or the first three major criteria with the minor criterion can be diagnosed with ET [10].

The ET-related complications include hemorrhagic, vasomotor symptoms and arterial/venous thrombosis in the absence of other risks like endocarditis [8, 9]. Treatment of ET depends on whether the patient is symptomatic or asymptomatic. Recommended drugs used are hydroxyurea, aspirin, anagrelide, and interferon-alpha [11, 12]. A thromboembolic phenomenon with MI has been documented in individuals with this mutation, but these patients are often symptomatic (chest pain), particularly when there are vascular involvements in high flow organs like the heart. Some patients develop MI after the diagnosis of ET [13]. In other cases, a patient may present with symptomatic chest pain and MI before diagnosis [14]. JAK2 V617F-positive patients with cardiovascular diseases do not have an increased predilection for future atheroembolism and routine screening for JAK2 mutation in such patients is not recommended except when they have concomitant elevated blood cell counts [15]. Yet, peripheral artery disease may predispose persons to the likelihood of JAK2 mutation [16]. Our patient presented with a chief complaint of abdominal pain, but the abnormal EKG was the first indication for an atypical MI. We believe the occlusion of the distal left anterior descending coronary artery led to an apical thrombus, which was the initial trigger for thrombosis and embolism to his right kidney and spleen. The apical thrombus was also the probable etiology for developing a stroke. We initially hypothesized that his initial elevated platelet count was a result of secondary reaction from MI but the continued rise in platelets was inconsistent with our hypothesis. Unfortunately, the patient refused a bone marrow biopsy and so, we were unable to diagnose him with ET using the WHO diagnostic criteria. Our case demonstrates that atypical MI with mild thrombocytosis may be the first manifestation of likely JAK2 V617F-positive ET.
